# Olfactory Mating Signals in the Migratory Locust *Locusta migratoria*

**DOI:** 10.1007/s10886-023-01456-9

**Published:** 2023-10-18

**Authors:** Anjana P. Unni, Markus Knaden, Bill S. Hansson

**Affiliations:** Max Planck Institute for Chemical Ecology, Hans Knoell Strasse 8, 07745 Jena, Germany

**Keywords:** Migratory locusts, Chemical ecology, Mating, Aggregation, Behavior

## Abstract

Swarming locusts cause huge plagues across the world threatening food production. Before swarms form, locust populations exhibit a dramatic phase change from a solitary to a gregarious phase. The cause of this phase change is a complicated interplay of conspecific and environmental cues and is, especially for one of the major pests, the migratory locust *Locusta migratoria*, still not well understood. Here we study the behavior of both solitary and gregarious *L. migratoria* towards the headspace odors of conspecifics. As we do not find a general attraction of gregarious animals to the headspace of gregarious conspecifics, swarm formation does not seem to be mainly governed by olfactory aggregation cues. When testing for potential mating signals, we observe that the headspace of virgin gregarious females is highly attractive only towards virgin males of the same phase, while mated gregarious males and solitary males, regardless of their mating state, do not become attracted. Interestingly, this phase-specific attraction goes along with the finding, that mating behavior in experiments with inter-phasic pairings is extremely rare. Our data suggest that odor emissions in *L. migratoria* play a significant role in a mating context.

**Supplementary Information:**

The online version contains supplementary material available at 10.1007/s10886-023-01456-9.

## Introduction

The devastating effect of swarms of gregarious locusts has been reported since biblical times. While solitary and gregarious locusts had been considered to be different species, in 1921, Uvarov discovered the fascinating phenomenon of phase polyphenism (Pflüger and Bräunig 2021; Simpson et al. 2011). As we now know from several locust species (Topaz et al. 2012), *Locusta migratoria* (Linnaeus 1758; Order: Orthoptera, Family: Acrididae) exists in solitary and gregarious phases. These phases differ in morphological, anatomical, and behavioral features (Greenwood and Chapman 1984; Latchininsky 2019; Wang et al. 2014; Wei et al. 2017), with e.g., the solitary phase being more camouflaged, while the gregarious phase is conspicuously colored. The shift between both phases is a complex phenomenon, that to some extent is still considered a puzzle. In desert locusts *Schistocerca gregaria* (Forsskål 1775; Order: Orthoptera, Family: Acrididae), factors such as environmental cues and sensory cues from conspecifics, including visual, tactile, and olfactory cues, seem to be involved (Nakano et al. 2022; Roessingh et al. 1998; Simpson et al. 2011). The role of olfaction in the locusts’ phase polyphenism has been explored over the past few decades (Guo et al. 2020; Wei et al. 2017). It is well understood that odor profiles are dynamic within the life stages and between the phases (Wei et al. 2017). Most of the existing literature, however, focuses on *S. gregaria* and suggests that headspace odors from gregarious animals are attractive to gregarious and repulsive to solitary conspecifics (Roessingh et al. 1993, 1998; Rogers et al. 2003). However, as *S. gregaria* and *L. migratoria* even differ in their responses to the pivotal body odor phenylacetonitrile (PAN) that is present in both species (Pener and Simpson 2009; Torto et al. 1996; Wei et al. 2019), the general function of headspace odors in *L. migratoria* so far remains elusive. Moreover, phase shift dynamics also differ directionally between the phases in *S. gregaria* (Simpson et al. 1999). However, there is a lack of understanding about *L. migratoria* that prompts further research to understand the behavioral response of *L. migratoria* to conspecific smells.

Here, we provide headspace odor collections of different developmental stages of gregarious *L. migratoria* to individual animals in a binary choice arena. Some studies have so far focused on the behavioral responses towards animal-released odors mainly in *S. gregaria* (Obeng-Ofori et al. 1993; Torto et al. 1996). We, therefore, performed a comprehensive set of experiments to test, whether stage-, phase-, and/or sex-specific odor blends provoke attraction or repulsion to the different stages, phases, and sexes in *L. migratoria*. At the same time, interphasic mating occurs in *S. gregaria* (Golov et al. 2018a) but this phenomenon has not been studied in *L. migratoria*. We, therefore, extend our study to mating assays between the different phases of *L. migratoria*. In conclusion, we aim to increase our understanding of *L. migratoria* with regard to the behavioral impact of its body odors.

## Methods and Materials

### Animal Breeding

We used *L. migratoria* that we bought from a local pet shop. The gregarious and solitary animals used for tests were kept separated for a minimum of 5 generations. Both phases were maintained at the Max Planck Institute for Chemical Ecology in climate chambers with a 14:10 h light:dark cycle, at a temperature of 30 ± 2 °C, and humidity of 50 ± 5%. The gregarious animals were kept with around 300–400 first instar animals in a cubic cage (side length, 30 cm × 30 cm × 30 cm). The numbers of animals were reduced continuously during aging, to finally reach around 200 adult animals in the same cage. The solitary animals were separated on the day of hatching into individual cylindrical boxes (height, 10.5 cm; diameter 8 cm). Each solitary animal was supplied with a separate ventilation system. Both phases were fed with wheat grass provided by our greenhouse.

### Bioassay Arena

**Fig. 1:**
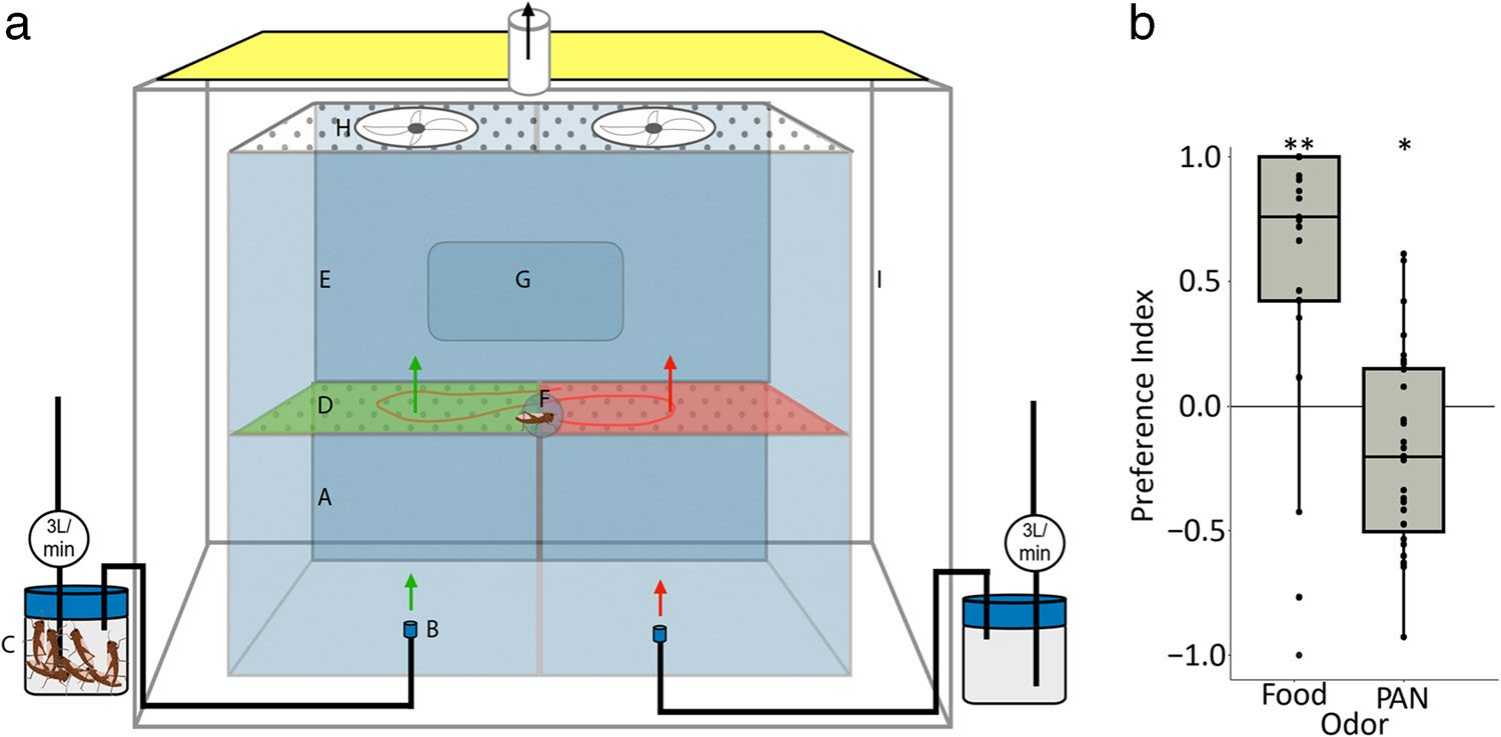
Assay for testing attraction and aversion of odors. **a** Experimental setup: A, polypropylene base; B, air diffusers; C, odor source; D, odor (green) and control (red) zones of arena; E, arena enclosure; F, opening to introduce animal; G, window to remove animal; H, axial fan; I, enclosing chamber. **b** Gregarious *L. migratoria nymphs* show significant attraction to food odors (*n* = 23, *p* = 0.0018, *Wilcoxon-signed rank test*) and repulsion to PAN (*n* = 31, *p* = 0.017, *Wilcoxon-signed rank test*). Box represents the 50% of the central data/interquartile range (IQR) with median, the whiskers represent the range (= upper quartile + 1.5 IQR/ lower quartile-1.5IQR). ***, *p* < 0.001; **, *p* < 0.01; *, *p* < 0.05

The behavioral setup includes a cuboid base, an arena surface consisting of a mesh, and an arena enclosure (Fig. 1a). The base of the behavioral setup consists of two separate polypropylene boxes (A) (16 cm × 30.5 cm × 25 cm) with air diffusers opening upwards at the middle of the lower surface (B) of each of the boxes. The diffuser is connected to the odor/control source (C). The base is high enough to evenly distribute the odor at the base before the air enters the arena. The in-house air is controlled by two flowmeters connected to the odor and control source via a 6/4 mm Teflon pipe. The air inlet is kept at 3L/min for each side. From the source, the air is introduced to the base of each side of the setup through the diffuser. Two perforated polypropylene plates of size 25 cm × 30 cm, with perforations of 2 mm diameter and distributed evenly at every 2 mm are placed on each of the two boxes making a rectangular behavioral arena (D). The arena has no division in the middle, allowing the locust to move in all directions within the arena. This design results in an arena divided into two zones, one with odor and one without that the animal can chose depending on the valence of the tested odor. The arena is enclosed by another rectangular polypropylene box (26 cm × 62 cm × 39 cm) to limit the locust within the arena (E). A circular inlet of 5 cm diameter that can be opened and closed from outside is situated at the same level as the behavioral arena plane to introduce locusts into the arena (F). A rectangular opening of 29 cm × 25 cm with a closing door is situated above the animal inlet to retrieve the animal after each trial, with the least disturbance to the airflow (G). The base, arena, and enclosing walls are supported with aluminum hinges of 1 cm width. A pair of axial fans (H) (connected to the same voltage input) are suspended on top of each side 39 cm above the arena to ensure a laminar and vertical airflow (ca. 3 cm/s) in the arena. Between the fans, an HD USB camera covering the whole arena is fitted and connected to a computer outside. The whole setup is placed inside an enclosing chamber 92*72*65 cm with solid white walls to exclude visual distraction from outside of the arena (I). LED light is fitted 11 cm above the enclosure to ensure even lighting throughout the arena surface. The air from the enclosure is ventilated out at a constant rate of approximately 30L/min via an exhaust fitted 15 cm above the behavioral setup. The setup is maintained at 31 ± 1°C and humidity at 50% during the trials.

### Bioassay Procedure

For each trial, one animal was introduced into the arena through the animal inlet, and each animal was tested only once. If an animal refused to enter the arena within the first 5 min, it was removed, and a new trial was started with a new animal. The 10-min recording of the animal’s behavior started only after the animal made an initial movement of at least 1 cm or more into the arena. After every 5 trials, the arena was wiped with 70% ethanol and ventilated for an hour with clean air to remove any odors and any potential trails left by the animals.

20 unstarved animals in an air-tight box were used as an odor source. After every 5 trials, the animals used as the odor source were placed back into the breeding cage for an hour after which they were again used as the odor source in the setup. In all tests, the side of odor within the arena was reversed after half of the trials. The nymphs used were all in the late fourth instar and not separated by males or females. The virgin animals for experiments were taken 6–8 days post eclosion and the mated animals were taken a few hours after the male debarks the female or the female finishes oviposition. The solitary animals were simply marked on the cage on the date of eclosion and used 8 days later for the tests.

### Mating Experiments

To measure the willingness to mate, we used mounting behavior by males (virgin animals 6–8 days after the final eclosion) as a parameter. The animals were kept together in the cage and were observed until the first mounting happened. The mounted pair was removed and the remaining animals were separated into males and females and used for experiments within 48 h of separation. Mating tests were done in a 10 cm × 10 cm cage built from perforated plates on 5 sides and a glass on one side allowing observation. Each cage, considered as one data point, consists of four males and five females. The proportion of the number of males mounting the females was observed at 6 and 12 h. This was done inter-phase as a test and intra-phase as control experiments.

### Data Analysis

In the odor choice bioassay procedure, the time (in seconds) an individual animal spent in the control or the odor side was observed. We considered the exact middle of the setup to be the division and the place of the head of the animal as the animal’s location when the animal spent time in the 2 cm-wide fringe area of the two zones.

The preference index was calculated as: $PI=(Time in Odor(s)-Time in Control(s))/(Total time (600s))$. To test whether the preference was significantly attractive or aversive, we used the *Wilcoxon-signed rank test*.

Mounting behavior was analyzed by the *Mann Whitney-U test*, at 6 h. To compare between multiple groups, at 12 h, *Kruskal–Wallis test with Dunn’s posthoc test* for multiple comparisons was used. In all cases, both for *PI* and mating experiments, ***, *p* < 0.001; **, *p* < 0.01; *, *p* < 0.05. *Wilcoxon-signed rank test* was performed in R version 4.2.3. For multiple group comparison GraphPad InStat was used.

PAN used for the experiments was ordered in the highest purity available from Sigma Aldrich (B19401-250G).

## Results

### Evaluation of Bioassay

To screen for the behavioral valence of body odors, we used a two-zone arena, where the headspace of 20 animals from a given stage was infused to one side, while the other side was infused with control air (Fig. 1a). The time spent by individual animals in either zone was measured to investigate whether the odor was perceived as attractive (i.e., more time spent on the odor side), or repellent (i.e., more time spent on the control side).

To test the assay for functionality, experiments were first performed with an attractive food odor (i.e., headspace emitted by 5 g of shredded wheat grass) and with the known repellent PAN (100 µL at 1 mg/mL concentration) diluted with mineral oil. PAN is repulsive in this assay to all stages and phase of the *L. migratoria* (Chang et al. 2023). When testing starved fourth instar gregarious nymphs with the food odor, the animals showed strong attraction to the odor and spent significantly more time on the side of the arena smelling of wheat (Fig. 1b). Animals of the same cohort tested with PAN avoided the side with this odor (Fig. 1b), demonstrating that the assay indeed was suitable for testing both attraction and aversion.

### Responses of Nymphs in Bioassay

**Fig. 2:**
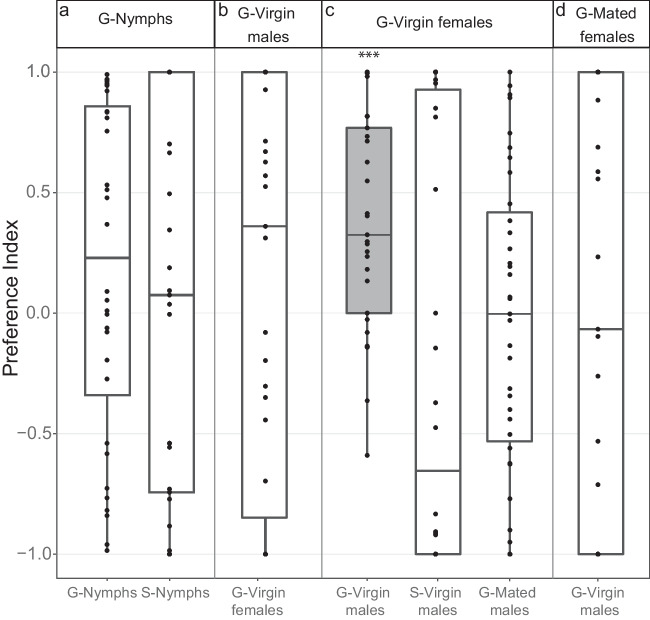
Attraction of animal headspaces in *L. migratoria*. **a** Preference of gregarious (*n* = 32, *p* = 0.13) and solitary nymphs (*n* = 29, *p* = 0.66) tested with headspace odors of gregarious nymphs. **b** Gregarious virgin females (*n* = 31, *p* = 0.50) tested with odors of gregarious virgin males. **c** Gregarious virgin males (*n* = 29, *p* = 0.0004), solitary virgin males (*n* = 30, *p* = 0.25), and gregarious mated males (*n* = 35, *p* = 0.74) tested with the odor of gregarious virgin females. **d** Gregarious virgin males (*n* = 27, *p* = 0.95) tested with odors of gregarious mated females. *Wilcoxon-signed rank test* was used to determine P values. Box represents the 50% of the central data/interquartile range (IQR) with median, the whiskers represent the range (= upper quartile + 1.5 IQR/ lower quartile-1.5IQR). ***, *p* < 0.001

Gregarious locusts usually aggregate in conspicuous huge hopper bands or swarms, whereas locusts of the solitary phase avoid groups and are rather cryptic. To investigate whether the aggregation and repulsion in gregarious and solitary animals, respectively, is governed by olfactory cues from the gregarious nymphs, the valence of odors from gregarious nymphs was tested in gregarious and solitary nymphs. Surprisingly, we did not find any significant response either in gregarious or in solitary nymphs to the headspace of gregarious nymphs (Fig. 2a). Similarly, no other tested headspaces from gregarious adults, except for the headspace of gregarious virgin females, elicited any significant attraction in gregarious or solitary nymphs (Supplementary Fig. 1). It thus seems that olfactory cues alone are not sufficient for the forming of hopper bands in gregarious *L. migratoria*.

### Responses of Adults in Bioassay

We next asked whether attraction towards potential mates might be governed by olfactory cues. When testing gregarious virgin males and virgin females with the headspace of the opposite sex, males were significantly attracted to the female odor, while females did not respond to the male odor (Fig. 2b, c). Interestingly, when testing for inter-phase attraction, we found that the odors from gregarious virgin females did not elicit any response in solitary virgin males (Fig. 2b). In addition, contrary to the headspace of virgin gregarious females, the odor emitted by mated gregarious females was not attractive to virgin gregarious males (Fig. 2d), suggesting that the headspace of mated females is either lacking attractive compounds or includes repellent compounds that virgin females are not emitting. At the same time, the attraction of gregarious males towards the odor of virgin gregarious females was diminished when the males were already mated (Fig. 2c).

### Mating Experiments

**Fig. 3:**
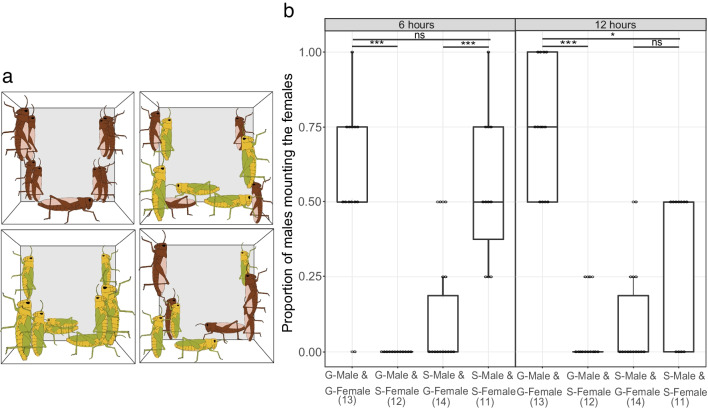
Intra- and inter-phase mating behavior in *L. migratoria*. **a** Schematic presentation of the mating experiment for an overview of the crowding of animals in the experiment. Cubic cage (side lengths 10 cm), experimental animals drawn to scale. The gregarious animals are represented in brown while the solitary animals are green and yellow. **b** Proportion of males mounting females in the intra- and inter-phase combinations of solitary and gregarious animals observed after 6 (left) and 12 (right) hours. *Kruskal–Wallis test with Dunn’s posthoc* test for selected comparisons. Box represents the 50% of the central data/interquartile range (IQR) with median, the whiskers represent the range (= upper quartile + 1.5 IQR/ lower quartile-1.5IQR). ***, *p* < 0.001; **, *p* < 0.01; *, *p* < 0.05

Having found that solitary males showed no attraction to the headspace of virgin gregarious females, we tested if this results in an inter-phase mating barrier. We conducted mating experiments, where virgin males were paired with either virgin females of the same or opposite phase (Fig. 3a). We then observed the mounting behavior which is an obligatory and easily observable step in orthopteran mating. Interestingly, we found that the frequency of mounting was significantly lower in inter-phase tests. At the end of 6 h, while 59% of the gregarious males mounted gregarious females, only 10% of the solitary males mounted the gregarious females. At the same time, 54% of the solitary males mounted the solitary females, while none of the gregarious males mounted solitary females during the 6 h of observations (Fig. 3b). While intra-phase combinations resulted in similar mounting ratios in gregarious and solitary animals during the first 6 h of the experiments, the mounting behavior of gregarious animals lasted longer, as many solitary males had already unmounted their solitary females by the end of 12 h (Fig. 3b).

## Discussion

The phase state in locusts has been shown to be governed both by environmental cues and cues from conspecifics (Nakano et al. 2022). From a sensory point of view, different developmental stages of *S. gregaria* aggregate based on both chemo- and mechanosensory cues (Niassy et al. 1999; Rogers et al. 2003; Simpson et al. 2001). Contact cues and short-range odors seem to be pivotal in the gregarious phase, while long range auditory cues are more important in the solitary phase (Nakano et al. 2022; Pener and Simpson 2009). The short-range odors emitted and perceived by the gregarious animals emanate from food, feces, and the headspace of conspecifics. Based on these results we expected, that gregarious nymphs of *L. migratoria*, which form hopper bands, are attracted by the headspace of gregarious nymphs. Surprisingly, we did not find any attraction, and gregarious nymphs in most aspects did not differ from solitary ones, which were neither attracted nor repulsed by any of the gregarious headspaces.

*S. gregaria* have been reported to shift easily from the solitary to the gregarious phase (Rogers et al. 2003), whereas for *L. migratoria*, the solitary phase seems to be the more stable state, and a shift to the gregarious phase is difficult to induce and needs an extremely high density of animals. Corresponding to that the rate of gregarization and solitarisation is also different within and between locust species (Topaz et al. 2012). *S. gregaria* exhibit a much faster gregarisation and a slower solitarisation process (Simpson et al 1999; Wei et al. 2017). A higher concentration of odors or even a combination of multi-modal cues including e.g., mechanosensory or visual signals might thus potentially be necessary to trigger aggregation behavior among *L. migratoria* nymphs.

The headspace composition has been shown to be dynamic and dependent on phase and state (Wei et al. 2017). 4-vinylanisole (4 VA) is one of the headspace odors that has been identified as a possible aggregation pheromone in *L. migratoria* (Guo et al. 2020). PAN is another dominant headspace odor in both *L. migratoria* and *S. gregaria*. The role of PAN in *S. gregaria* is still under debate, as some studies suggest a role as an aggregation pheromone (Torto et al. 1994), others supposed repellency (Seidelmann et al. 2005), while yet others even suggest a role in sexual behavior (Seidelmann and Ferenz 2002). In *L. migratoria*, however, PAN is repulsive to animals of all stages and phases (Chang et al. 2023; Wei et al. 2019). The responses of nymphs that we observed in the aggregation tests could thus be expected, considering the odor profile of individual groups. The higher proportion of PAN to 4VA in gregarious males as compared to gregarious females (Wei et al 2019) could form a background to the neutral response to gregarious male odors, where the attractant 4VA and the repellent PAN balance each other. The strongly attractive response by gregarious nymphs to gregarious females could rely on the higher amount of 4VA, which would dominate over the repellency of PAN. Similar mixture interactions in binary mixtures of opposing valence have also been reported in e.g., *Drosophila* (Mohamed et al. 2019; Thoma et al. 2014).

When looking for potential effects of headspace odors on mating behavior, we found a strong attraction of gregarious males to the headspace of virgin gregarious females. As the number of gregarious males is much higher than that of gregarious females in mating swarms and in ovipositing populations (Ellis and Ashall 1957), gregarious males face strong competition for females. The indifferent response of the virgin gregarious females to the headspace of virgin gregarious males is coherent with this proportion, as females do not have to actively respond to males for successful mating. The lack of response of virgin gregarious males to mated females could indicate the presence of a courtship inhibitory factor. Such a factor can be either produced by the female herself to prevent further harassment from males (Engel et al. 2016) or be transferred by the male during mating as an additional passive form of mate guarding (Seidelmann 2006). Male-transferred anti-aphrodisiacs are found in other insects too (Khallaf et al. 2020, vander Meer et al. 1986). In *S. gregaria*, PAN acts as such a courtship inhibition pheromone (Seidelmann and Ferenz 2002). Alternatively, mated females could emit lower amounts of pheromones. The lack of response by mated gregarious males to the odor of virgin gregarious females could be interpreted as a sign of transient abstinence during a recovery phase (Barrozo et al. 2010a) which has been shown in other insects to be combined with a lower sensitivity to female pheromones post mating (Barrozo et al. 2010b).

When testing mating behavior within the phases, we already found differences between the gregarious and the solitary animals. The mounting of solitary males usually lasted for less than 12 h, while that of gregarious males lasted much longer. Mounting of females by males is the longest copulation step and is present in both phases. It has been proposed as an active mate guarding strategy of the male to avoid remating of the female before his sperm has fertilized her eggs (Golov et al. 2018a; Zhu and Tanaka 2002). The duration of the mounting varies depending on sub species, duration of separation of males into cohorts, and phase (Golov et al. 2018a, b; Seidelmann 2006; Tanaka and Zhu 2003). The longer mounting that we observed in the gregarious phase could, hence, be interpreted as an effect of the higher male-male competition faced by gregarious males within the swarm.

In the inter-phase attraction experiments, we found it intriguing that solitary males were not attracted by the headspace of virgin gregarious females. We, therefore, asked whether males and females from different phases would mate at all. In *S. gregaria* gregarious males exhibit frequent mounting attempts when encountering solitary females. Interestingly, we found an opposite trend in *L. migratoria*. Males of a given phase showed only weak interest in females of the other.

In conclusion, we found that odors emitted by nymphs seem to be of less importance in attracting other nymphs into hopper bands. Other sensory cues or odors of a higher concentration, or combinations of these might be the deciding factors. The attraction between the sexes does, however, seem to be relying on female-produced cues as gregarious males were strongly attracted to the odor of virgin gregarious females. Interestingly, solitary males were not attracted to the smell of gregarious females, revealing a certain degree of an inter-phase mating barrier. This postulation was further corroborated by our finding that male mounting behavior in couples of mixed phases is very rare.

### Supplementary Information

Below is the link to the electronic supplementary material.

Supplementary file1 (DOCX 93 KB)

## Data Availability

Data will be made available on request.

## References

[CR1] Barrozo RB, Gadenne C, Anton S (2010). Post-mating sexual abstinence in a male moth. Commun Integr Biol.

[CR2] Barrozo RB, Gadenne C, Anton S (2010). Switching attraction to inhibition: mating-induced reversed role of sex pheromone in an insect. J Exp Biol.

[CR3] Chang H, Cassau S, Krieger J (2023). A chemical defense deters cannibalism in migratory locusts. Science.

[CR4] Ellis PE, Ashall C (1957). Field studies on diurnal behaviour, movement and aggregation in the desert locust (*Schistocerca gregaria* Forskål). Anti-Locust Bull.

[CR5] Engel KC, Stökl J, Schweizer R (2016). A hormone-related female anti-aphrodisiac signals temporary infertility and causes sexual abstinence to synchronize parental care. Nat Commun.

[CR6] Forsskål P (1775) Descriptiones animalium, avium, amphibiorum, piscium, insectorum, vermium; quae in itinere orientali observavit Petrus Forsskål. Post mortem auctoris edidit Carsten Niebuhr. Havenai, Mölleri, p 164

[CR7] Golov Y, Harari A, Rillich J, Ayali A (2018). Precopulatory behavior and sexual conflict in the desert locust. PeerJ.

[CR8] Golov Y, Rillich J, Douek M (2018). Sexual behavior of the desert locust during intra-and inter-phase interactions. J Insect Behav.

[CR9] Greenwood M, Chapman RF (1984). Differences in numbers of sensilla on the antennae of solitarious and gregarious *Locusta migratoria* L. (Orthoptera: Acrididae). Int J Insect Morphol Embryol.

[CR10] Guo X, Yu Q, Chen D (2020). 4-Vinylanisole is an aggregation pheromone in locusts. Nature.

[CR11] Khallaf MA, Auer TO, Grabe V, Depetris-Chauvin A, Ammagarahalli B, Zhang DD, Lavista-Llanos S, Kaftan F, Weißflog J, Matzkin LM, Rollmann SM, Löfstedt C, Svatoš A, Dweck HKM, Sachse S, Benton R, Hansson BS, Knaden M (2020). Mate discrimination among subspecies through a conserved olfactory pathway. Sci Adv.

[CR12] Latchininsky AV (2019). Locusts. Encycl Anim Behav.

[CR13] Linnaeus C (1758) Systema naturae per regna tria naturae, secundum classes, ordines, genera, species, cum characteribus, differentiis, synonymis, locis. Tomus I. Editio decima, reformata 1:824

[CR14] Mohamed AAM, Retzke T, Das Chakraborty S (2019). Odor mixtures of opposing valence unveil inter-glomerular crosstalk in the *Drosophila* antennal lobe. Nat Comm.

[CR15] Nakano M, Morgan-Richards M, Trewick SA, Clavijo-McCormick A (2022). Chemical ecology and olfaction in short-horned grasshoppers (Orthoptera: Acrididae). J Chem Ecol.

[CR16] Niassy A, Torto B, Njagi PGN (1999). Intra- and interspecific aggregation responses of *Locusta migratoria migratorioides* and *Schistocerca gregaria* and a comparison of their pheromone emissions. J Chem Ecol.

[CR17] Obeng-Ofori D, Torto B, Hassanali A (1993). Evidence for mediation of two releaser pheromones in the aggregation behavior of the gregarious desert locust, *Schistocerca gregaria* (forskal) (Orthoptera: Acrididae). J Chem Ecol.

[CR18] Pener MP, Simpson SJ (2009). Locust phase polyphenism: An update. Adv Insect Phys.

[CR19] Pflüger HJ, Bräunig P (2021). One hundred years of phase polymorphism research in locusts. J Comp Physiol A.

[CR20] Roessingh P, Simpson SJ, James S (1993). Analysis of phase-related changes in behaviour of desert locust nymphs. Proc Royal Soc B.

[CR21] Roessingh P, Bouaïchi A, Simpson SJ (1998). Effects of sensory stimuli on the behavioural phase state of the desert locust*, **Schistocerca gregaria*. J Insect Physiol.

[CR22] Rogers SM, Matheson T, Despland E (2003). Mechanosensory-induced behavioural gregarization in the desert locust *Schistocerca gregaria*. J Exp Biol.

[CR23] Seidelmann K (2006). The courtship-inhibiting pheromone is ignored by female-deprived gregarious desert locust males. Biol Lett.

[CR24] Seidelmann K, Ferenz HJ (2002). Courtship inhibition pheromone in desert locusts, *Schistocerca gregaria*. J Insect Physiol.

[CR25] Seidelmann K, Warnstorff K, Ferenz H-J (2005). Phenylacetonitrile is a male specific repellent in gregarious desert locusts, *Schistocerca gregaria*. Chemoecology.

[CR26] Simpson SJ, McCaffery A, Hägele BF (1999). A behavioural analysis of phase change in the desert locust. Biol Rev.

[CR27] Simpson SJ, Despland E, Hägele BF, Dodgson T (2001). Gregarious behavior in desert locusts is evoked by touching their back legs. Proc Natl Acad Sci USA.

[CR28] Simpson SJ, Sword GA, Lo N (2011). Polyphenism in insects. Curr Biol.

[CR29] Tanaka S, Zhu DH (2003). Phase-related differences in mating strategy of a locust (Orthoptera: Acrididae). Ann Entomol Soc Am.

[CR30] Thoma M, Hansson BS, Knaden M (2014). Compound valence is conserved in binary odor mixtures in *Drosophila melanogaster*. J Exp Biol.

[CR31] Topaz CM, D’Orsogna MR, Edelstein-Keshet L, Bernoff AJ (2012) Locust dynamics: Behavioral phase change and swarming. PLoS Comput Biol 8:e1002642 10.1371/journal.pcbi.1002642 PMC3420939 22916003

[CR32] Torto B, Obeng-Ofori D, Njagi PGN (1994). Aggregation pheromone system of adult gregarious desert locust *Schistocerca gregaria* (forskal). J Chem Ecol.

[CR33] Torto B, Njagi PGN, Hassanali A, Amiani H (1996). Aggregation pheromone system of nymphal gregarious desert locust, *Schistocerca gregaria* (Forskål). J Chem Ecol.

[CR34] vander Meer RK, Obin MS, Zawistowski S (1986). A reevaluation of the role of cis-vaccenyl acetate, cis-vaccenol and esterase 6 in the regulation of mated female sexual attractiveness in *Drosophila melanogaster*. J Insect Physiol.

[CR35] Wang X, Fang X, Yang P (2014). The locust genome provides insight into swarm formation and long-distance flight. Nat Comm.

[CR36] Wei J, Shao W, Wang X (2017). Composition and emission dynamics of migratory locust volatiles in response to changes in developmental stages and population density. Insect Sci.

[CR37] Wei J, Shao W, Cao M (2019). Phenylacetonitrile in locusts facilitates an antipredator defense by acting as an olfactory aposematic signal and cyanide precursor. Sci Adv.

[CR38] Zhu DH, Tanaka S (2002). Prolonged precopulatory mounting increases the length of copulation and sperm precedence in *Locusta migratoria* (Orthoptera: Acrididae). Ann Entomol Soc Am.

