# Correction: Olfactory Mating Signals in the Migratory Locust *Locusta Migratoria*

**DOI:** 10.1007/s10886-024-01487-w

**Published:** 2024-03-15

**Authors:** Anjana P. Unni, Markus Knaden, Bill S. Hansson

**Affiliations:** Max Planck Institute for Chemical Ecology, Hans Knoell Strasse 8, 07745 Jena, Germany

**Correction: Journal of Chemical Ecology**

10.1007/s10886-023-01456-9

The published online proof of this paper is incorrect and not the updated one as it still contains the watermark “Uncorrected Proof”.

The original article has been corrected.

