# Supplementary material for: Olfactory Mating Signals in the Migratory Locust Locusta migratoria
Source: J Chem Ecol. 2023 Oct 18;50(1-2):11–7. doi: 10.1007/s10886-023-01456-9 (PMC10991065; doi:10.1007/s10886-023-01456-9)
Supplement: Supplementary file 1 — Supplementary file1 (DOCX 93 KB) [file 10886_2023_1456_MOESM1_ESM.docx]

SUPPLEMENTARY DATA


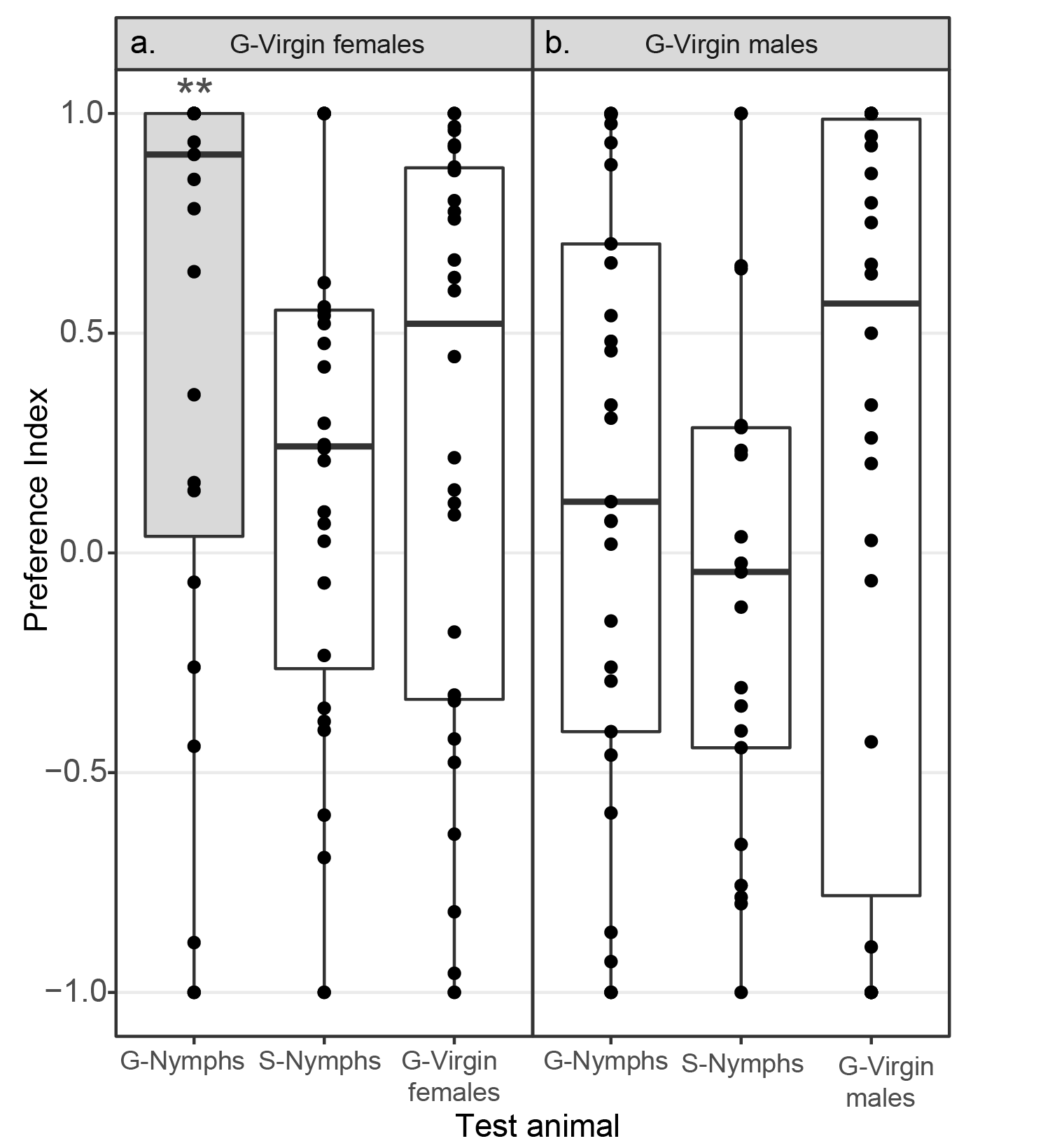


Supplementary Figure 1- a. Attraction of *L. migratoria* gregarious nymphs (n=27, p=0.008), solitary nymphs (n=28, p=0.11) and gregarious virgin females (n=30, p=0.07) tested against virgin gregarious female odors. b. The response of gregarious nymphs (n=29, p=0.33), solitary nymphs (n=21, p=0.48), and gregarious virgin males (n=30, p=0.29) to the gregarious virgin male odors. The *Wilcoxon-signed rank test* was used to determine P values. Box represents the 50% of the central data/interquartile range (IQR) with median, the whiskers represent the range (=upper quartile+1.5 IQR/ lower quartile-1.5IQR). ***, p<0.001; **, p<0.01; *, p<0.05.
